# Genome-wide association analysis of cardiovascular-related quantitative traits in the Framingham Heart Study

**DOI:** 10.1186/1753-6561-3-s7-s117

**Published:** 2009-12-15

**Authors:** Nicole M Roslin, Jemila S Hamid, Andrew D Paterson, Joseph Beyene

**Affiliations:** 1 Program in Genetics and Genome Biology, MaRS Centre, The Hospital for Sick Children Research Institute, 101 College Street, Toronto, Ontario M5G 1L7, Canada; 2 Biostatistics Methodology Unit, Child Health Evaluative Sciences, The Hospital for Sick Children Research Institute, 123 Edward Street, Toronto, Ontario M5G 1E2, Canada; 3 Dalla Lana School of Public Health, University of Toronto, 155 College Street, Toronto, Ontario M5T 3M7, Canada

## Abstract

Multivariate linear growth curves were used to model high-density lipoprotein (HDL), low-density lipoprotein (LDL), triglycerides (TG), and systolic blood pressure (SBP) measured during four exams from 1659 independent individuals from the Framingham Heart Study. The slopes and intercepts from each of two phenotype models were tested for association with 348,053 autosomal single-nucleotide polymorphisms from the Affymetrix Gene Chip 500 k set. Three regions were associated with LDL intercept, TG slope, and SBP intercept (*p *< 1.44 × 10^-7^). We observed results consistent with previously reported associations between rs599839, on chromosome 1p13, and LDL. We note that the association is significant with LDL intercept but not slope. Markers on chromosome 17q25 were associated with TG slope, and a single-nucleotide polymorphism on chromosome 7p11 was associated with SBP intercept. Growth curve models can be used to gain more insight on the relationships between SNPs and traits than traditional association analysis when longitudinal data has been collected. The power to detect association with changes over time may be limited if the subjects are not followed over a long enough time period.

## Background

Cardiovascular disease (CVD) is the leading cause of death in the United States and is a significant cause of disability. Worldwide, heart disease is on the rise, and is predicted to become the leading cause of death and disability by 2020 [1].

In order to identify common risk factors leading to CVD, several large-scale epidemiological studies have been undertaken. Most notable is the Framingham Heart Study (FHS), a prospective study which was started over 50 years ago and is still ongoing. It was designed to follow the development of CVD over time in a large group of participants who had not yet developed symptoms of CVD. The original participants underwent extensive medical testing approximately every 2 years, and more recently recruited individuals have also been followed regularly. Through the data arising from the FHS, several major risk factors for CVD have been identified, including hypertension, high blood cholesterol, smoking, obesity, and diabetes. In addition, results from the study have demonstrated significant effects of demographic factors such as age and sex.

The longitudinal design of the FHS allows for the study of how certain traits change over time. The analysis of time-dependent data can range from simple plots to complex survival or multilevel modelling. In this paper, we use a latent growth curve (LGC) model to examine the change over time in levels of systolic blood pressure (SBP), high-density lipoprotein (HDL), low-density lipoprotein (LDL), and triglycerides (TG), as well as to explore the relationship between these four traits, which are known to affect the risk of developing CVD. Association analysis was performed to identify genetic factors that are associated with mean baseline values of each trait, as well as the changes over time, using subjects from the FHS.

## Methods

Details about sample recruitment can be found in Cupples et al. [2] and Splansky et al. [3]. Briefly, 5209 subjects aged 29 to 62 were recruited between 1948 and 1953 from the town of Framingham, Massachusetts (Original Cohort). Between 1971 and 1975, an additional 5124 individuals were recruited, who were the offspring of the Original Cohort and the offspring's spouses (Offspring Cohort). Finally, between 2002 and 2005, 4095 third generation individuals (children of the Offspring Cohort) were recruited (Generation 3). Data from four examinations are available for each of the Original and Offspring Cohort, while data from a single examination are available for Generation 3.

### Samples used for analysis

Because individuals from the Original Cohort fasted before only one of the four exams, the lipid profiles obtained from these individuals were not ideal for longitudinal analyses. We therefore restricted our analysis to members of the Offspring Cohort, who fasted before all four exams. We selected independent members of the Offspring Cohort as follows. Starting with the original 1538 families, the Generation 3 cohort was removed, which split the pedigrees into 3379 independent sub-pedigrees. Individuals belonging to the Offspring Cohort who had phenotype and genotype data were considered for inclusion in the analysis. We selected independent individuals from this set. In sub-pedigrees in which multiple sets of individuals could be chosen, we randomly selected a set that gave the largest number of independent individuals (as determined by the kinship coefficient). This resulted in 1488 individuals. An additional 171 samples without family data were added, for a total of 1659 independent individuals.

### Phenotypic modeling

A linear LGC model was fit to longitudinal measurements of SBP, HDL, LDL, and TG. One of the strengths of LGC modeling is that it allows the study of multiple outcomes over time in a multivariate framework, which is particularly useful in investigating the change in phenotype values and assessing cross-phenotype relationships [4]. Two models were analyzed, corresponding to two sets of covariates. In the first set, sex, baseline age, and body mass index (BMI), and a variable to indicate a diagnosis of diabetes at any time during the study were included as time-invariant covariates, and the number of cigarettes smoked per day was considered to be a time-varying covariate. The second covariate set was identical, except that BMI was allowed to vary over time. In both models, individuals who reported taking medication at the time of examination had their relevant trait values adjusted by the addition of a constant. This procedure has been shown to provide good power to detect a genetic effect and to have little bias in effect size estimation, while being relatively robust to the exact value of the constant [5]. For individuals taking medication for hypertension, SBP was increased by 10 mm Hg [6]. For individuals taking lipid-lowering medication, the reported HDL values were adjusted by -2.15 mg/dL, LDL by +43.23 mg/dL, and TG by +24.92 mg/dL, based on previously reported average effects of statins and fibric acid derivatives in primarily White subjects [7]. Because information on the type of medication was not provided for our sample, the effects of the two types of drugs were combined using a weighted average. Missing values were imputed using the missing-at-random assumption. The models were fit using the software Mplus version 5 [8]. Because the distribution of TG was skewed, the robust maximum-likelihood method was used. LDL was calculated for each observation using the Friedewald equation [9], or was considered to be missing if TG>400 mg/dL.

### Genotypes

Genotyping was conducted using the Affymetrix GeneChip Human Mapping 500 k Array Set, using the 250 k Sty and 250 k Nsp platforms. Only autosomal markers were considered for analysis. The original set of markers consisted of 487,014 autosomal markers with known chromosomal assignments and physical positions. We removed 32,594 (6.7%) markers where the call rate was <95%, based on all 6848 genotyped individuals. Markers were also removed if the minor allele frequency was <5% (101,422 markers) or the *p*-value from an exact test for Hardy-Weinberg equilibrium [10] was <10^-6 ^(4945 markers). Thus, the final marker set consisted of 348,053 single-nucleotide polymorphisms (SNPs).

### Association analysis

Individual-specific intercepts and slopes were obtained from the growth curve models, for each of the phenotypes SBP, HDL, LDL, and TG. These phenotypic summaries were examined for association with each marker separately using linear regression, as implemented in the program PLINK v1.04 [11], assuming an additive genetic model. Markers associated with slope traits affect the change over time of a particular phenotype, while markers associated with intercept traits affect the initial value.

## Results and discussion

### Description of phenotypes and covariates

The sample was composed of 1659 independent subjects who were examined at four different time points. Details about the raw variables and covariates are described in Table 1, before and after adjustment for medication use. The amount of missing phenotypic data varied between exams. Exam 1 was the most complete, with a missing rate of 0.7% for the variables used in this study. Exam 3 was the least complete, with a missing rate of 13.9%, mostly due to 208 individuals who did not attend this examination. Exams 5 and 7 had missing rates of 6.3% and 5.0%, respectively. Summaries of the slopes and intercepts obtained after fitting the growth curve models to HDL, LDL, TG, and SBP are shown in Table 2. With the exception of TG, the trait values did not change much over the four time points, as indicated by the small slopes. The time-invariant covariates age, sex and diabetes were significant at the 5% level for the intercept of all traits, but not necessarily the slopes (data not shown). Comparing the model in which BMI was treated as a time-invariant covariate with the model in which BMI was allowed to vary over time, the distributions of the estimates were shifted in location for all traits, but the variances were largely unchanged.

**Table 1:** Sample characteristics^a^

Variable	Exam 1	Exam 3	Exam 5	Exam 7
Examined^b^	1659 (100)^a^	1451 (87.5)	1563 (94.2)	1597 (96.3)
Exam dates	1971 to 1975	1983 to 1987	1991 to 1995	1998 to 2001
Males	754 (45.4)	667 (46.0)	711 (45.5)	724 (45.3)
Age, years	34.8 ± 9.0	47.3 ± 9.0	54.3 ± 8.9	61.0 ± 8.9
BMI, kg/m^2^	25.0 ± 4.1	25.9 ± 4.6	27.2 ± 4.7	27.9 ± 5.2
SBP, mm Hg	118.9 ± 14.0 *119.1 ± 14.2*^d^	122.3 ± 16.0 *123.6 ± 17.3*	124.7 ± 18.7 *126.3 ± 20.0*	126.3 ± 18.8 *129.4 ± 20.5*
Hypertension medication	30 (1.8)	189 (13.0)	251 (16.1)	502 (31.4)
HDL, mg/dL	51.5 ± 14.4 *51.5 ± 14.4*	52.0 ± 14.8 *52.0 ± 14.8*	50.5 ± 15.0 *50.4 ± 15.1*	54.5 ± 17.0 *54.1 ± 17.2*
LDL, mg/dL	123.2 ± 34.3 *123.4 ± 34.7*	136.7 ± 36.3 *137.1 ± 36.9*	126.3 ± 32.4 *129.4 ± 35.3*	119.8 ± 31.6 *128.5 ± 32.8*
TG, mg/dL	83.6 ± 62.7 *83.7 ± 62.2*	114.4 ± 128.4 *114.6 ± 128.6*	145.4 ± 119.3 *147.3 ± 120.3*	135.8 ± 86.8 *140.8 ± 88.8*
Cholesterol medication	7 (0.4)	14 (1.0)	117 (7.5)	321 (20.1)
Smokers	654 (39.6)	380 (26.2)	269 (17.2)	206 (12.9)
Diabetes^c^	175 (10.5)			

^a^Values are mean ± standard deviation for quantitative variables, and count (%) for qualitative variables.

^b^Individuals with a recorded age at exam.

^c^Individuals who developed diabetes at any point during the study.

^d^Values in italics are means and standard deviations after adjustment for medication use.

**Table 2:** Characteristics of the intercepts and slopes from the growth curve models^a^

	BMI time-invariant^b^			BMI time-varying^c^		
		
Trait	Intercept	Slope	Slope/intercept ratio (%)	Intercept	Slope	Slope/intercept ratio (%)
HDL	51.6 ± 10.9	0.37 ± 2.20	0.72	74.9 ± 9.9	1.75 ± 2.24	2.33
LDL	126.4 ± 29.0	1.36 ± 5.66	1.07	85.6 ± 28.2	7.85 ± 5.18	9.17
TG	83.2 ± 50.7	20.86 ± 20.41	25.03	-16.4 ± 48.8	12.31 ± 20.97	74.92
SBP	120.3 ± 10.8	3.08 ± 3.31	2.56	93.5 ± 9.2	0.09 ± 3.25	0.10

^a^Values are mean ± standard deviation. Units are mg/dL for HDL, LDL, and TG intercept; mm Hg for SBP intercept; mg/dL per visit for HDL, LDL, and TG slope; mm Hg per visit for SBP intercept.

^b^Model included sex, baseline age, diabetes status, and baseline BMI as time-invariant covariates, and number of cigarettes smoked per day as a time-varying covariate.

^c^Model identical to BMI time-invariant model, except BMI was treated as a time-varying covariate.

### Association analysis

We tested 348,053 markers for association with the intercepts and slopes of HDL, LDL, TG, and SBP. A total of six markers in three regions were associated in at least one of the two models, using a Bonferroni cutoff of *p *< 1.44 × 10^-7 ^(Table 3). The most significant association was between LDL intercept and rs599839 on chromosome 1p13 (*p *= 3.04 × 10^-10 ^in the model where BMI was time-varying). The minor G allele was associated with a reduction in LDL intercept values. This marker is approximately 250 kb upstream of *PSRC1*, which encodes a proline-rich protein. This marker was recently shown to be associated with LDL measured at a single examination in a meta-analysis [12], and was also associated with coronary artery disease [13]. In these two studies, the major A allele was associated with an increase in LDL levels, or risk of coronary artery disease, consistent with the direction we report here. Although this SNP was associated with LDL intercept, it was not associated with LDL slope (*p *= 0.14), indicating that it may not play a role in the change of LDL levels over time, at least in the age range of individuals studied here. This SNP also showed the most significant association with LDL intercept in the model in which BMI was time-invariant (*p *= 2.62 × 10^-9^).

**Table 3:** Association results for markers with *p *< 1.44 × 10^-7^

				BMI time-invariant^a^		BMI time-varying^b^	
					
Trait	Locus	Marker	Minor allele (frequency)	(SE)^c^	*p*-Value	(SE)^c^	*p*-Value
LDL intercept	1p13	rs599839	G (0.23)	-7.14 (1.19)	2.62 × 10^-9^	-7.33 (1.16)	3.04 × 10^-10^
TG slope	17q25	rs6501683	T (0.23)	4.73 (0.86)	3.89 × 10^-8^	5.25 (0.88)	2.98 × 10^-9^
	17q25	rs7213663	C (0.24)	4.26 (0.84)	4.98 × 10^-7^	4.68 (0.87)	7.47 × 10^-8^
	17q25	rs8074960	C (0.24)	4.31 (0.84)	3.27 × 10^-7^	4.71 (0.86)	5.51 × 10^-8^
	17q25	rs8075297	C (0.24)	4.17 (0.83)	5.30 × 10^-7^	4.57 (0.85)	8.99 × 10^-8^
SBP intercept	7p11	rs11976165	T (0.20)	2.53 (0.47)	6.88 × 10^-8^	2.11 (0.40)	1.45 × 10^-7^

^a^Phenotypic model included sex, baseline age, diabetes status and baseline BMI as time-invariant covariates, and number of cigarettes smoked per day as a time-varying covariate.

^b^Phenotypic model identical to BMI time-invariant model, except BMI was treated as a time-varying covariate.

^c^ represents the regression coefficient from the association analysis, and estimates the effect on the trait value by adding one copy of the minor allele; SE, standard error.

SNP rs6501683 on 17q25 was associated with TG slope (*p *= 3.89 × 10^-8 ^and 2.98 × 10^-9 ^for BMI time-invariant and BMI time-varying models, respectively). Three other markers in the same region also show association, although at lower levels of significance. All four markers are in nearly complete linkage disequilibrium (*r*^2 ^= 0.99 or 1 for all pairs of markers) in this data. TG intercept was not associated with these markers (*p *= 0.19 and 0.20 for BMI time-invariant and BMI time-varying models, respectively, for rs6501683).

On chromosome 7p11, rs11976165 was associated with SBP intercept (*p *= 6.88 × 10^-8^) in the model in which BMI was treated as a time-invariant covariate. In the model in which BMI was treated as a time-varying covariate, the *p*-value for this SNP falls just above the significance cutoff used here (*p *= 1.45 × 10^-7^). This region is peri-centromeric, and is not near any known genes. The observed association was only with the intercept (*p *= 0.20 for SBP slope, BMI time-invariant model).

Because there may be genes in common to the change in BMI, lipid levels, and SBP, two models were fit differing only in the treatment of the covariate BMI. One model was fit using baseline BMI as a covariate, and another in which BMI was included as a time-varying covariate. The model in which BMI was time-invariant may be better able to detect association with regions affecting both BMI and the traits of interest. However, in the dataset used here, the association results for the two models were very similar.

In order to account for the effect of trait-altering medications, we adjusted medicated values by adding a constant to the relevant observations. Adjusting for medication use by including it as a time-varying covariate in the model did not change the association patterns between the traits and markers, although a slight shrinkage bias was observed in the effect size estimates (data not shown). Because only a small proportion of individuals reported taking trait-altering medication, particularly in Exams 1, 3, and 5, we did not expect the choice of medication adjustment method to have a strong effect on the overall results.

Because the distribution of TG was non-normal, we used robust maximum-likelihood estimation [14], as implemented in the Mplus statistical software [8]. The distributions of the resulting intercepts and slopes were less skewed, but still showed a long upper tail, which could affect inference of the association tests. Additionally, the distribution of the TG intercepts from the model in which BMI was time-varying tended to be negative. This may indicate that the departure from normality was too extreme to be modeled well by robust techniques, or that a linear trajectory was not an appropriate assumption. A log transformation could normalize the distribution, at the cost of decreased interpretability of the estimates.

We did not explicitly model time, as measured by age at exam, in the growth model. This implicitly assumes that the exams were evenly spaced, an assumption which seems reasonable, on average, between Exams 3, 5, and 7, but not between Exams 1 and 3. The model could be improved by including a more appropriate measure of time.

The estimates of the slopes tended to be small for all traits, with the exception of TG. Thus, in this sample, the trait values tended to remain stable over time, and perhaps it is not surprising that significant association with a slope trait was only observed with TG. The heritability of the intercepts and slopes from this study cannot be calculated because independent samples were used. However, other analyses of the FHS data showed that the heritability of lipid levels taken at a single exam is moderate (*h*^2 ^= 0.52, 0.59, and 0.48 for HDL, LDL, and TG, respectively) [15], while the heritability of SBP at a single exam is lower (*h*^2 ^= 0.28) [16]. Studies investigating the heritability of the change in phenotypes over time are less common, although the heritability of the SBP slope was estimated to be similar to that of a single exam (*h*^2 ^= 0.23) in the FHS data [17].

## Conclusion

The longitudinal nature of the FHS data was exploited using LGC models, which allowed the study of multiple phenotypes simultaneously. Consequently, the effect of each phenotype on each other was accounted for, through pairwise correlations, in the model. An association between a marker and a particular trait, therefore, can be interpreted as an association with the trait, after the effects of the covariates and the remaining three traits have been accounted for. We used phenotypic summaries from these models to search for SNPs associated with the mean value or change over time of lipid and blood pressure phenotypes across the genome. Because long-term averages of lipid phenotypes have been shown to be heritable [16], this strategy may allow us to distinguish genes contributing to overall levels of traits from those contributing to changes over time.

## List of abbreviations used

BMI: Body mass index; CVD: Cardiovascular disease; FHS: Framingham Heart Study; HDL: High-density lipoprotein; LDL: Low-density lipoprotein; LGC: Latent growth curve; SBP: Systolic blood pressure; SNP: Single-nucleotide polymorphism; TG: Triglyceride.

## Competing interests

The authors declare that they have no competing interests.

## Authors' contributions

NMR participated in the design of the study, performed the association analysis, and drafted the manuscript. JSH participated in the design of the study, performed the growth curve modeling, and helped to draft the manuscript. ADP participated in the biological interpretation of the data and analysis results. JB participated in the design of the study. All authors read and approved the final manuscript.
